# Using Perfusion Contrast for Spatial Normalization of ASL MRI Images in a Pediatric Craniosynostosis Population

**DOI:** 10.3389/fnins.2021.698007

**Published:** 2021-07-19

**Authors:** Catherine A. de Planque, Henk J. M. M. Mutsaerts, Vera C. Keil, Nicole S. Erler, Marjolein H. G. Dremmen, Irene M. J. Mathijssen, Jan Petr

**Affiliations:** ^1^Department of Plastic and Reconstructive Surgery and Hand Surgery, University Medical Center Rotterdam, Rotterdam, Netherlands; ^2^Department of Radiology and Nuclear Medicine, Amsterdam Neuroscience, Amsterdam University Medical Center, Amsterdam, Netherlands; ^3^Department of Biostatistics, University Medical Center Rotterdam, Rotterdam, Netherlands; ^4^Department of Epidemiology, Erasmus Medical Center, Rotterdam, Netherlands; ^5^Department of Radiology and Nuclear Medicine, University Medical Center Rotterdam, Rotterdam, Netherlands; ^6^Helmholtz-Zentrum Dresden-Rossendorf, Institute of Radiopharmaceutical Cancer Research, Dresden, Germany

**Keywords:** ASL, segmentation, registration, spatial normalization, pediatric, craniosynostosis

## Abstract

Spatial normalization is an important step for group image processing and evaluation of mean brain perfusion in anatomical regions using arterial spin labeling (ASL) MRI and is typically performed via high-resolution structural brain scans. However, structural segmentation and/or spatial normalization to standard space is complicated when gray-white matter contrast in structural images is low due to ongoing myelination in newborns and infants. This problem is of particularly clinical relevance for imaging infants with inborn or acquired disorders that impair normal brain development. We investigated whether the ASL MRI perfusion contrast is a viable alternative for spatial normalization, using a pseudo-continuous ASL acquired using a 1.5 T MRI unit (GE Healthcare). Four approaches have been compared: (1) using the structural image contrast, or perfusion contrast with (2) rigid, (3) affine, and (4) nonlinear transformations – in 16 healthy controls [median age 0.83 years, inter-quartile range (IQR) ± 0.56] and 36 trigonocephaly patients (median age 0.50 years, IQR ± 0.30) – a non-syndromic type of craniosynostosis. Performance was compared quantitatively using the real-valued Tanimoto coefficient (TC), visually by three blinded readers, and eventually by the impact on regional cerebral blood flow (CBF) values. For both patients and controls, nonlinear registration using perfusion contrast showed the highest TC, at 17.51 (CI 6.66–49.38) times more likely to have a higher rating and 17.45–18.88 ml/100 g/min higher CBF compared with the standard normalization. Using perfusion-based contrast improved spatial normalization compared with the use of structural images, significantly affected the regional CBF, and may open up new possibilities for future large pediatric ASL brain studies.

## Introduction

Spatial normalization is an important step for brain image processing; it not only enables group analyses but is also required for automatic segmentation of tissue type and brain regions. Functional or physiological MRI acquisitions, such as arterial spin labeling (ASL) perfusion MRI, typically perform nonlinear registration via conventional structural – mostly T1-weighted (T1w) – scans for their higher resolution and structural contrast. However, in situations where the tissue contrast is low and changing, such as in early phases of myelination in newborns and infants, these structural reference scans may not help or even fail normalization (Knickmeyer et al., 2008; Dubois et al., 2014; Holland et al., 2014).

The use of other images with higher tissue-contrast could help registration. As an alternative to spatial normalization via segmentation and registration of structural images, studies use contrast from different MRI modalities. Feng et al. (2019) used Diffusion Tensor Imaging (DTI) as a substitute for T1w scans. In DTI images, premyelination is encountered prior to being detectable at T1w or T2w imaging (Yoshida et al., 2013). Similarly, Mutsaerts et al. (2018) used cerebral blood flow (CBF) and pseudo-CBF, created from a gray matter (GM) map from segmented T1w image to register individual ASL and T1w volumes instead of using the morphological images for the registration, for example, the ASL control images or M0 scans registered to T1w images in elderly subjects. This approach was especially important in cases where the image contrast difference between GM and white matter (WM) was low in ASL control images or in M0 scans, due to, for example, use of strong background suppression or short TR, respectively. This approach can be potentially extended to direct spatial normalization of ASL to standard space in the pediatric population as ASL studies of the brain show sufficient CBF contrast between GM and WM already in early age despite the potential lack of GM/WM contrast in T1w images (Yoshida et al., 2013).

The problem with spatial normalization in subjects with ongoing myelination is of particular clinical relevance in imaging babies with inborn or acquired disorders impairing normal brain development. Craniosynostosis, referred to the premature fusion of the skull sutures leading to skull and brain deformations, is an example of such disease (de Jong et al., 2010; Johnson and Wilkie, 2011; Maliepaard et al., 2014; Spruijt et al., 2015). Trigonocephaly, a non-syndromic type of craniosynostosis that presents within a sliding scale of severity in phenotype and brain imaging, is one of the key components in the decision making for surgical treatment in the first years of life. Imaging of these newborns is essential, and ASL is an MRI technique that could provide cerebral perfusion measurements on both a global and regional level.

Spatial normalization is then necessary to be able to evaluate perfusion in predefined anatomical regions. However, automatic methods for spatial normalization are challenging in young children with craniosynostosis as there are issues with low GM/WM contrast and skull deformity, asexplained above (de Jong et al., 2010; Keil et al., 2019). In previous ASL studies in craniosynostosis patients, regions of interest (ROI) were therefore placed manually, which made spatial normalization escapable (Doerga et al., 2019). However, this practice is time consuming, and it increases the likelihood of error in delineation and decreases the repeatability across subjects.

To overcome the issues with spatial normalization of T1w images, we propose to use different contrast than from the structural images for the spatial normalization. In this study, we set to investigate if the ASL CBF image contrast can be directly used for spatial normalization in children with trigonocephaly and healthy controls under the age of 18 months. We combined technical and clinical expertise to compare the standard method that uses structural images for the normalization with three different registrations of ASL directly to MNI using rigid, affine, and nonlinear transformations. We hypothesize that direct ASL spatial normalization to the MNI space is possible, that nonlinear registration can be used in this context to improve the normalization quality in young healthy controls and trigonocephaly patients, and that this normalization will have a significant effect on the measured regional CBF. With this study we aim to facilitate the investigation of frontal lobe perfusion in trigonocephaly patients in a clinical setting to assess the value of vault surgery in these patients.

## Materials and Methods

The Ethics Committee approved this prospective imaging study in patients with trigonocephaly (METC-2018-124), which is part of ongoing work at the Erasmus Medical Center involving protocolized care, brain imaging, clinical assessment, data summary, and evaluation (de Planque et al., 2021). To participate in this study, informed research consent has been obtained.

### Subjects

Preoperative MRI brain scans from 36 children with metopic synostosis for whom a surgical correction was considered were included over a period of 2 years (2018–2020). Surgery was considered only for moderate and severe presentation of metopic synostosis, mainly defined by severe narrowing and a protruding midline ridge of the forehead, hypotelorism (eyes close together), and biparietal widening (Birgfeld et al., 2013). Children were less than 2 years of age at the time of the MRI brain study. The control group consisted of sixteen subjects undergoing MRI brain studies for clinical reasons, with the following inclusion criteria: (1) no neurological pathology of the head or neck (e.g., children with intracranial masses, prior neurosurgeries, known myelin disorders); (2) no neurological or psychological morbidity on follow-up; and (3) no residual motion artifacts in the subjects’ brain MRI data.

### MRI Acquisition

All MRI data were acquired using a 1.5 Tesla MR Unit (General Electric Healthcare, Milwaukee, WI, United States), and the imaging protocol included a three-dimensional spoiled gradient T1w MR sequence. Imaging parameters included slice thickness of 2 mm, no slice gap, a field of view 22.4 cm, matrix size 224 × 224, in plane resolution 1 mm, echo time 3.1 ms and repetition time 9.9 ms. The pseudo-continuous ASL sequences with the following imaging parameters: 3D fast spin-echo spiral readout with a stack of 8 spirals and 3 averages, repetition time 4,604 ms, echo time 10.7 ms, voxel size 3.75 mm × 3.75 mm × 4.0 mm, field of view 24.0 cm × 24.0 cm, labeling duration 1,450 ms, post-labeling delay 1,025 ms, and background suppression. This protocol was identical in both trigonocephaly patients and controls. Both groups underwent deep sedation or sevoflurane-induced anesthesia during the MRI procedure.

### Image Processing

Data processing and analysis were performed with ExploreASL, a Matlab-based toolbox (MathWorks, MA, United States) developed to facilitate quality control and analyses for single or multicenter ASL studies (Mutsaerts et al., 2014, 2015, 2020). This toolbox is based on Statistical Parametric Mapping (SPM) 12 (Wellcome Trust Centre for Neuroimaging, University College London, United Kingdom; Ashburner, 2007).

The preprocessing included the default 3D ASL processing of ExploreASL except for the ASL-T1w registration part, which includes the quantification of the M0 and CBF image (Mutsaerts et al., 2020).

Afterward, we compared four approaches: (1) normalization via segmentation and spatial normalization of the T1w images (regT1); a direct registration of ASL to MNI using either (2) rigid-body (regASLrigid), (3) affine (regASLaffine), or (4) a nonlinear Direct Cosine Transform (DCT) transformation (regASLdct).

For regT1, the T1w image was segmented – to GM, WM, and cerebrospinal fluid – and registered to MNI space with ExploreASL default settings except for using SPM12 with the NITRC 2.3 brain template of 1-year-olds. The 1-year-old template was chosen for all subjects, since this was the closest match for the majority of our subjects (Shi et al., 2011). The M0 and T1w images were rigidly aligned and applied to ASL to align it with T1w. The spatial normalization of ASL for regT1 was then obtained as the joint transformation of ASL to T1w and T1w to MNI space.

For the regASLrigid, regASLaffine, regASLdct, a pseudo-CBF image was constructed in the MNI space using the GM and WM probability maps from the NITRC template resampled to 1.5 mm × 1.5 mm × 1.5 mm voxels, multiplying them by 60 and 20 ml/min/100 g, respectively, and finally smoothing them to the ASL resolution. The native ASL CBF image was then registered with the pseudo-CBF image using the respective transformations. The nonlinear DCT consisted of 16 discrete-cosine basis functions along each dimension (Ashburner and Friston, 1999). For all the abovementioned transformations, all registration steps were combined in a single joint transformation with a single interpolation from native to standard space.

### Quantitative Evaluation

We compared the four normalization methods quantitatively by studying the overlap between the individual CBF image registered to the standard space and a CBF template in the standard space using the Tanimoto similarity coefficient for real-valued vectors, see equation (1) in the work of Anastasiu and Karypis (2017). The Tanimoto coefficient (TC) is a measure of image overlap, ranging from 0% (completely dissimilar) to 100% (identical images). If we assume that perfect registration does not lead to identical images but ones that still retain physiological differences, TC >70% can be regarded as excellent image agreement. We masked the brain in both images and normalized the values in each image to the 97th percentile value while excluding the higher signal values from the computation. Note that we computed the whole-brain TC for continuous perfusion-weighted values (also known as Tanimoto distance) rather than the commonly used measure for binary images (Anastasiu and Karypis, 2017).

### Qualitative Evaluation

The spatial normalization of ASL images to the MNI space was inspected qualitatively by examining the overlay of CBF and outer WM borders in MNI space with a threshold at 50% WM partial volume. The T1w-based and the three types of ASL-based normalizations were visually scored by three raters: a pediatric neuroradiologist (MD, 7 years of radiology experience), an ASL image processing engineer (JP, 10 years of experience), and a neuroradiologist (VK, 10 years of experience).

The visual alignment of the ASL images in MNI space was categorized into four quality categories: (1) unusable, 2) poor, (3) usable, and (4) excellent. For the evaluation of the results of each normalization method of each subject, the rater rated overviews of normalizations showing 12 axial and 12 sagittal slices. All visual overlay images from both the patient and control groups for all four normalization methods were pseudo-randomized and pseudonymized. The pseudonym was visible to the raters throughout the whole procedure of individual rating and for reaching consensus. The raters were blinded to the method and clinical history of the patients and controls.

### Statistical Analyses

Statistical analyses were performed using R (Version 4.0.3; R Core Team, 2020). The descriptive characteristics are presented as mean and SD or as median and inter-quartile range (IQR), depending on whether the data are normally distributed or not. Categorical data are presented as counts. Age differences between patients and controls were assessed using Student’s *t*-test. A chi-square test was performed to assess the effect of sex between groups. A significance level of 0.05 was chosen for all tests.

Differences in TC between patients and controls were investigated for each of the normalizations (T1w, rigid-body, affine, and DCT) using a scatterplot and a boxplot.

In addition, to investigate the differences in the consensus rating of different types of normalization, we fitted a Bayesian proportional odds cumulative logit mixed model. Besides the type of registration, the model included the patient’s group (patient vs. control) and random intercepts for each subject to take into account the fact that different types of normalization performed in the same child are likely correlated. The results are presented as odds ratios and corresponding 95% credible intervals (CI). To facilitate interpretation of the estimated differences in ratings between the different types of normalization, we plotted the estimated probability of receiving a particular rating for each type of normalization (in patients). To compare the mean CBF in three gyri of the frontal lobe in trigonocephaly patients vs. controls for different normalizations, we fitted a mixed linear model for each of the gyri. A random intercept was included to take into account the fact that measurements of different gyri in the same child are likely correlated. Mean regional CBF that was assessed in an anatomical region of interest cannot be reliably assessed in group analyses in children for whom the spatial normalization failed. To avoid bias in the CBF analysis, we have excluded all children from whom one of the four methods failed to generate CBF map in standard space or from whom the map was rated as unusable in the qualitative analysis. Due to the small sample size, no other variables could be included in the model.

## Results

### Characteristics

A total of 36 patients with trigonocephaly with a median age of 0.50 years (IQR 0.30) and sixteen control subjects with a median age of 0.83 years (IQR 0.56) were included (Table 1). The age was shown to be significantly different (*p* = 0.006) between patients (mean: 0.60 years) and controls (mean: 0.90 years). Sex was also significantly different between groups (*p* = 0.03), but had no significant effect on the frontal lobe perfusion in this cohort (*p* = 0.09).

**TABLE 1:** Patient characteristics.

	**Trigonocephaly (*n* = 36)**	**Controls (*n* = 16)**
Sex (*n* female)	11 (30.6%)	10 (62.5%)
Age (median ± IQR years)	0.50 ± 0.30	0.83 ± 0.56

### Quantitative Comparison of Normalization Strategies

**FIGURE 1:**
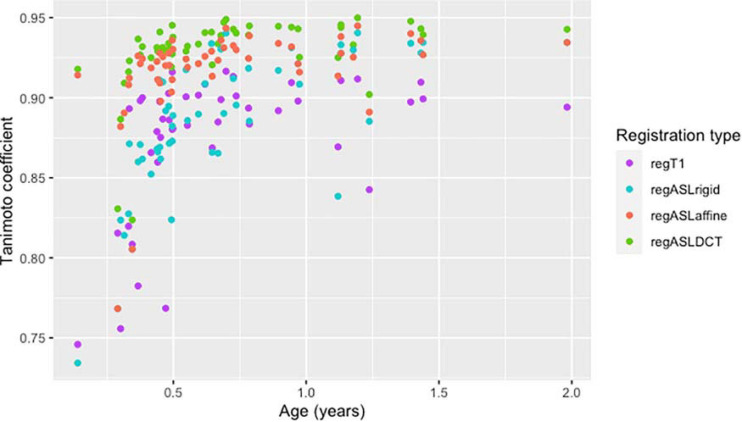
A scatterplot of the Tanimoto coefficient of four registration types of the total cohort in time (age in years).

**FIGURE 2:**
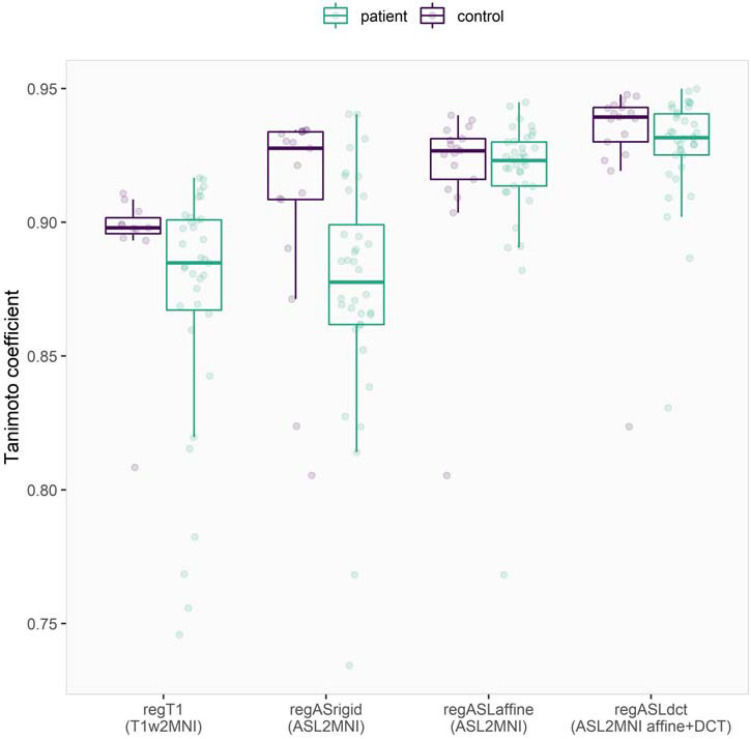
Visualization of the Tanimoto coefficient of the four registration types in patients and controls. The dots represent the original data, while the boxplots show the 25, 50, and 75% quantiles. The whiskers reach to a maximum of 1.5 times the IQR.

From all normalization strategies, regASLdct shows the highest TC score (Figure 1). The range of the TC is depicted for patients and controls in Figure 2. The overlapping boxplots of the ASL normalization types in the control group, demonstrate that there is little difference between these normalizations with respect to the TC. RegASLdct had the highest TC outcome for patients as well as controls. For patients, regASLaffine had better TC outcomes than regT1, or regASLrigid, whereas for controls regASLrigid performed similar to regASLaffine.

### Qualitative Comparison of Normalization Strategies

**FIGURE 3:**
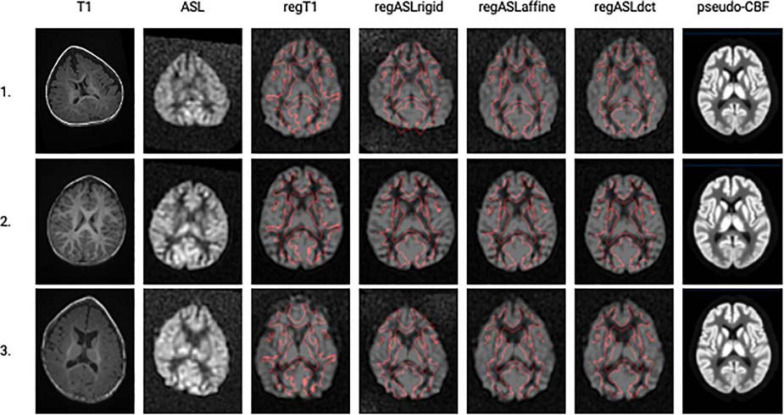
A single axial slice is shown (columns left to right) for the T1-weighted image and the ASL image in their native space before registration and for the results of the four registration methods in the standard space: regT1, regASLrigid, regASLaffine, and regASLdct of three trigonocephaly subjects (rows). In the standard space, a CBF image is shown with borders of the WM from the template (with a threshold at 50%) shown in red. Finally, the pseudo-CBF images of the three trigonocephaly patients are shown.

The T1w image, the ASL image, the four normalizations, and the pseudo-image of three trigonocephaly subjects are shown in Figure 3. As depicted in Table 2, the MRI of a patient with trigonocephaly received higher ratings compared with the MRIs of controls (OR 2.61 CI 0.70–9.95). RegASLrigid has lower odds of receiving a higher rating (OR 0.33 CI 0.14–0.77). The odds of having a higher rating are 2.61 times as large for regASLaffine (CI 1.11–6.25) and 17.51 times as large as for regASLdct (CI 6.66–49.38) compared with the odds of the regT1.

**TABLE 2:** Odds ratios for receiving a higher rating [i.e., the odds are calculated as *P*(rating > *k*)/*P*(rating = *k*)].

	**Odds ratio**	**2.5%**	**97.5%**
Trigonocephaly	2.611	0.698	9.954
RegASLrigid	0.332	0.143	0.770
RegASLaffine	2.608	1.113	6.249
RegASLdct	17.508	6.659	49.378

**FIGURE 4:**
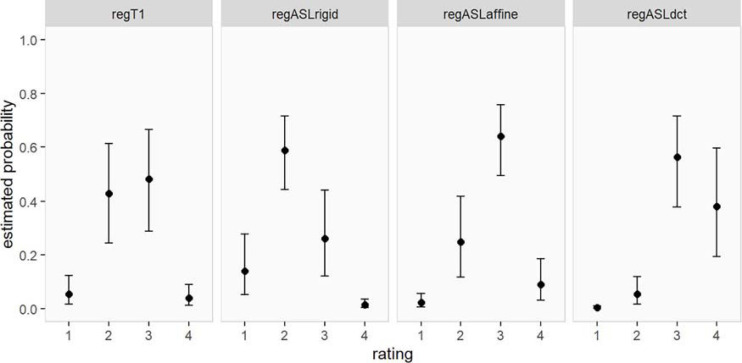
Estimated probability (and corresponding 95% CI) of obtaining a certain rating for each type of registration (for patients).

The resulting estimated probabilities for an MRI of a patient to receive a particular rating are visualized in Figure 4 and reported in Table 3. The highest probabilities of a good rating were estimated for regASLdct (0.38, 95% CI 0.19–0.60 for rating 4 and 0.56, 95% CI 0.38–0.72 for rating 3). RegASLaffine had the highest probability of receiving a rating of 3 (0.64, 95% CI 0.49–0.76), and a one in four chance of receiving a rating of 2 (95% CI 0.12–0.42). For regT1 and regASLrigid ratings 2 and 3 were most likely (regT1 rating 3: 0.48, 95% CI 0.29–0.66, rating 2: 0.43, 95% CI 0.24–0.61; regASLrigid rating 3: 0.26, 95% CI 0.12–0.44, rating 2: 0.59, 95% CI 0.44–0.72).

**TABLE 3:** Estimated probability of getting a particular rating for each type of registration (and corresponding 95% CI).

**Rating**	**RegT1**	**RegASLrigid**	**RegASLaffine**	**RegASLdct**
1	0.05 [0.02, 0.12]	0.14 [0.05, 0.28]	0.02 [0.01, 0.05]	0.00 [0.00, 0.01]
2	0.43 [0.24, 0.61]	0.59 [0.44, 0.72]	0.25 [0.12, 0.42]	0.05 [0.02, 0.12]
3	0.48 [0.29, 0.66]	0.26 [0.12, 0.44]	0.64 [0.49, 0.76]	0.56 [0.38, 0.72]
4	0.04 [0.01, 0.09]	0.01 [0.00, 0.03]	0.09 [0.03, 0.19]	0.38 [0.19, 0.60]

### CBF Comparison of Normalization Strategies

The results of the mixed linear model demonstrate that there was no evidence of a difference in the mean CBF of the frontal lobe between trigonocephaly patients and controls (Table 4). Moreover, the mean CBF evaluated in the frontal lobe was significantly higher for direct ASL registration to the MNI space via a pseudoCBF for all three types of registration (regASLrigid, regASLaffine, regASLdct) compared with regT1.

**TABLE 4:** Linear mixed model for the three gyri of the frontal lobes of 41 subjects using the Hammers Atlas (ml/100 g/min).

**Inferior frontal gyrus**	**Value**	**Standard error**	**2.5%**	**97.5%**
(Intercept)	52.81	4.68	43.54	62.08
Trigonocephaly patient	8.13	5.34	–2.71	18.97
RegASLrigid	9.33	0.90	7.55	11.12
RegASLaffine	17.11	0.90	15.32	18.89
RegASLdct	17.45	0.90	15.67	19.24
Middle frontal gyrus				
(Intercept)	50.15	5.07	40.09	60.21
Trigonocephaly patient	1.93	5.78	–9.82	13.67
RegASLrigid	10.59	1.05	8.51	12.68
RegASLaffine	18.94	1.05	16.86	21.02
RegASLdct	18.49	1.05	16.40	20.57
Superior frontal gyrus				
(Intercept)	45.75	4.63	36.58	54.93
Trigonocephaly patient	9.39	5.27	–1.32	20.09
RegASLrigid	14.28	1.00	12.29	16.26
RegASLaffine	18.99	1.00	17.01	20.98
RegASLdct	18.88	1.00	16.90	20.87

## Discussion

In this study, we have shown that direct normalization of ASL images to MNI space using ASL CBF as image contrast outperforms spatial normalization based on T1w segmentation in MRI brain studies of both patients and controls who are less than 2 years of age. The nonlinear registration outperformed both rigid and affine registration among the methods using the ASL CBF contrast. While better results in TC of the regASLdct were shown for the control group, the difference between regT1 and regASLdct was even higher in patients in both qualitative analysis and CBF analysis. The CBF values in three gyri of the frontal lobe, which are clinically relevant for trigonocephaly patients, were significantly different compared with the CBF values extracted using the spatial normalization with the regT1 method. This shows the impact of the choice of registration contrast and the importance of this proposed method in a cohort where gray-white matter contrast in structural images is low due to ongoing myelination.

Using the SPM-based segmentation and normalization pipeline in ExploreASL with T1w, our initial attempts to register and segment the brain of trigonocephaly patients showed poor performance, even though a dedicated template for young children was used (de Planque et al., 2021). Our normalization was complicated by the ongoing myelination in these young patients. Here, we were able to combine our previously developed CBF-contrast-based registration with a low-degree-of-freedom nonlinear component to improve the registration for the deformed skulls reaching a better registration (Mutsaerts et al., 2018). In this previous study of Mutsaerts et al., T1w images of elderly patients with frontotemporal dementia were spatially normalized to MNI and ASL was aligned with T1w images using the CBF-GM contrast. The joint transformation of ASL to T1w and T1w to MNI space was then used for spatial normalization of ASL to MNI. In the current study, we extended the previous work by aligning ASL to MNI directly using the CBF vs. GM contrast and investigating, also, the DCT transformation to assess the qualitative and quantitative benefit of this method in a pediatric patient cohort with skull deformations.

Both regT1 and regASLrigid performed poorly on the TC and qualitatively. For the regT1 we attribute this to the relatively low WM-GM contrast of these images. The poor performance of regASLrigid is likely caused by the fact that brain size and shape differ with age. Also, it is challenging to register with MNI with only a rigid registration that preserves size. The regASLaffine and regASLdct registrations show a better alignment. RegASLaffine and regASLdct addresses both issues and therefore performed better in TC and on the qualitative rating. The advantage of regASLdct over regASLaffine is that it can adapt to the nonlinear deformations present in the trigonocephaly patients. Therefore, rigid and affine registrations followed by nonlinear deformations using a linear combination of three-dimensional DCT basis functions were considered sufficient for alignment of the individual data to the template (Ashburner and Friston, 1999). The quality of the pseudo-CBF image is essential for the registration. Typical values of CBF of 60 and 20 ml/min/100 g in GM and WM, respectively, were used. Even though these values are only rough approximations and the population and individual values differ largely, they still capture the general differences in contrast between the tissue types, and this approximation was sufficient for the regASLrigid and regASLaffine registration. This, however, was not sufficient for the regASLdct registration with more degrees of freedom. To obtain good results, the regASLdct registration had to be performed in two iterations, where the regional CBF in GM and WM were first approximated from the ASL image after a rough alignment and used to construct a more realistic pseudo-CBF image in the second iteration. While the spatial normalization reached a good quality in most subjects, further improvement could be achieved in the future in large populations using population-specific templates. To address the relatively high deformation variability, the Cerebromatics approach could be used to create a template that covers a range of deformation types and severity. Subgroups other than trigonocephaly patients could also possibly be considered. To address more severe deformations, further deformation algorithms might need to be tested that provide potentially a better performance than the DCT registration (Klein et al., 2009).

Ground truth measurement of CBF was not present in this study to ultimately prove the validity of the proposed method for spatial normalization. However, since all methods contained the same preprocessing except for the spatial normalization, we assume that a difference in extracted regional CBF is a sufficient measure of the difference between the methods. Moreover, we assume that the method that performed better at the qualitative and quantitative evaluation of the spatial normalization will also be closer to the ideal normalization.

As the registration of spatial MRI sequences to structural regT1 images of the brain is a major challenge in patients with unmyelinated brains (such as neonates), there are other example studies concerning the use of contrast that is not usual for this population. Because many immature white matter tracts cannot be differentiated with conventional MR images (T1w or T2w) caused by insufficient myelination during the preterm development period, Feng et al. (2019) and Yoshida et al. (2013) used DTI as a substitute for T1w in a preterm population. Another example, which is also relevant for neonates, is tract-based spatial statistics, permitting a voxel-wise statistical analysis of the entire white matter skeleton instead of the usual DTI ROI approach (Smith et al., 2006; Duerden et al., 2015). This study could be an initial impetus for future large cohort pediatric ASL studies using ASL as an unusual contrast.

Imaging of preterm and term-born infants for clinical indications is of great interest. It has been demonstrated that various injuries, due to perinatal risk often leads to damage in selective white matter (Rutherford et al., 2004). De Vis et al. (2013) have already demonstrated that CBF progressively increases during the first period of life as synaptogenesis, myelination, and brain functional activity progress. Miranda et al. (2006) have observed cerebral perfusion was significantly higher in preterm newborns studied at term-corrected age than in term-born newborns, indicating that brain perfusion may be influenced by developmental and postnatal age. Both studies were based on manual ROI delineations of cortical regions or on manual placement of control points in GM and WM. Using the proposed normalization method provides clinical users an automatic delineation, which could potentially lead to a higher reproducibility and regional accuracy.

### Limitations

One main limitation is that we tested whole-brain alignment only, i.e., we have not systematically tested the alignment quality on the level of individual cortical structures. While the method proved to provide usable or excellent spatial normalization and thus should allow the reliabe assessment of perfusion in specific regions of the MNI space, a more thorough validation is needed to demonstrate whether this normalization method allows regional CBF evaluation in MNI space using gray and white matter masks. Currently, the method thus allows the study of regional brain perfusion even in the absence of a T1w image, but further validation is needed to fully replace T1w images in ASL processing when not available. For that reason, more advanced analysis that uses T2-weighted and DTI MR brain images to aid segmentation is planned. Moreover, we aim to study the GM CBF after partial volume correction. However, currently, tissue segmentation from T1w images is not possible for all subjects, which is a hurdle for use of partial-volume correction on an individual basis (Feng et al., 2019). Second, while this cohort of 36 preoperative MRI scans of trigonocephaly patients is limited in size, it is the first study on automated ASL evaluation in trigonocephaly patients aged 0–3 years. The craniofacial unit of the Erasmus MC continues with the prospective collection of preoperative MRI scans of craniosynostosis patients for clinical and research perspectives which includes also other patients than those with trigonocephaly. A validation of this methodology on a larger cohort is thus planned, which will include also patients with more severe skull deformations. A third limitation is that our control group consisted of patients who underwent MRI examination for clinical reasons, where the MRI and clinical course showed no cerebral pathology. At last, patients and controls were not age exactly-matched. While this might slightly affect the CBF difference and the performance of the normalization between groups, the key findings of this study lie in comparing normalization methods within each group for which this minor age difference bears no importance.

## Conclusion

In conclusion, when the conventional T1w and T2w contrast between GM and WM are hard to differentiate, spatial normalization is feasible by using the ASL perfusion contrast directly. The choice of contrast for registration has an impact on both the quality of ASL alignment and the extracted regional CBF values. The results of this study may be an important step toward the feasibility of future large pediatric ASL MRI brain studies.

## Data Availability Statement

The datasets presented in this article are not readily available because the raw data used in this study are subject to the European data protection policy (GPDR), hence will not be made publicly available. For access to the anonymized data, contact the corresponding author. All data we processed using ExploreASL version 1.4.0. This is publicly available at https://zenodo.org/record/4449854. All relevant settings are described in the Materials and Methods section. Requests to access the datasets should be directed to JP, j.petr@hzdr.de.

## Ethics Statement

The studies involving human participants were reviewed and approved by the Ethics Committee of the Erasmus Medical Center. Written informed consent to participate in this study was provided by the participants’ legal guardian/next of kin.

## Author Contributions

CP: conceptualization, data curation, formal analysis, investigation, methodology, project administration, resources, validation, writing – original draft, and writing – review and editing. HM: methodology, software, and supervision. VK: validation, writing – review and editing. NE: statistics. IM: conceptualization, funding acquisition, supervision, validation, and writing – review and editing. JP: supervision, validation, methodology, software, writing – original draft, and writing – review and editing. All authors contributed to the article and approved the submitted version.

## Conflict of Interest

The authors declare that the research was conducted in the absence of any commercial or financial relationships that could be construed as a potential conflict of interest.
